# Genome Sequencing Outperforms Exome Sequencing in Formalin‐Fixed Paraffin Embedded Tumors Leading to Improved Treatment Options

**DOI:** 10.1002/cam4.72448

**Published:** 2026-10-27

**Authors:** Nadja Ballin, Alexander Ott, Michaela Pogoda, Florian Harmuth, Vijay Kumar Ulaganathan, Anton Karelin, Cristiana Roggia, Irina Bonzheim, Stephan Singer, Holly Sundberg‐Malek, Pavlos Missios, Stephan Ossowski, Tobias B. Haack, Olaf Riess, Michael Bitzer, Nisar Malek, Christopher Schroeder, Sorin Armeanu‐Ebinger

**Affiliations:** ^1^ Institute of Medical Genetics and Applied Genomics University Hospital Tübingen Tübingen Germany; ^2^ NGS Competence Center Tübingen (NCCT) Tübingen Germany; ^3^ Department of Pathology and Neuropathology University Hospital Tübingen Tübingen Germany; ^4^ Center for Personalized Medicine University Hospital Tübingen Tübingen Germany; ^5^ Department of Internal Medicine I University Hospital Tübingen Tübingen Germany

**Keywords:** FFPE, molecular tumor board, precision oncology, tumor exome sequencing, tumor genome sequencing

## Abstract

Precision oncology is based on comprehensive molecular characterization of a patient's tumor. This is mostly realized by next‐generation sequencing panels or exome sequencing. Data on the value of genome sequencing from formalin‐fixed paraffin embedded (FFPE) material in a clinical setting is still scarce. In this pilot, we generated datasets of matched exome and genome sequencing of FFPE tumor samples from 15 cancer patients to benchmark quality metrics, clinical feasibility, and added value for a molecular tumor board. Despite the reduced sequencing depth, diagnostic detection of small nucleotide variations, copy number variations, and complex biomarkers was maintained in genome sequencing compared to exome sequencing with the exception of hard‐to‐sequence regions. Evaluation by the local molecular tumor board identified additional treatment recommendations with high priority in 20% (3/15) of patients and additional molecular markers to support existing treatment recommendations in another 20% (3/15) of patients. Our work underscores the diagnostic superiority of genome sequencing even for FFPE samples and therefore supports its integration into clinical practice.

**Abbreviations**
AMP: amplification
CNV: copy number variation
ES: exome sequencing
FF: fresh frozen
FFPE: formalin‐fixed paraffin embedded
GS: genome sequencing
het DEL: heterozygous deletion
hom DEL: homozygous deletion
HRD: homologous recombination deficiency
LOH: loss of heterozygosity
MSI: microsatellite instability
MTB: molecular tumor board
QC: quality control
SNV: single nucleotide variant
SV: structural variation
TMB: tumor mutation burden
VAF: variant allele frequency

## 1 Introduction

In the era of precision oncology, next‐generation sequencing (NGS)‐based detection of tumor‐specific oncogenic alterations has become important to select patients for targeted therapies. Tumor sequencing results are discussed in interdisciplinary molecular tumor boards and subsequent recommendations of personalized treatment strategies or suitable clinical trials are made [1]. Currently, this is primarily achieved through specialized panels or exome sequencing (ES), which covers all coding sequences, representing approximately 1% of the entire human genome [2]. Ideally, however, the underlying tumor analyses should be as broad as possible. Genome sequencing (GS) offers the most comprehensive analyses of genetic alterations today. Moreover, an added diagnostic value of GS compared to ES or other NGS‐based panel analyses has been reported in rare diseases and in solid tumors [3, 4, 5, 6, 7, 8]. From a technical point of view, GS has also been shown to be superior to ES due to enhanced coverage uniformity, detection of copy number variations (CNVs), and structural variations (SVs) [9, 10]. However, these require high‐quality DNA either isolated from peripheral blood or from fresh‐frozen (FF) tumor tissue. In routine clinical practice, tumor samples are in the vast majority available as formalin‐fixed and paraffin embedded (FFPE) material owing to its widespread use in pathology for long‐term preservation and histological assessment [11]. The fixation process can lead to DNA fragmentation, reduced yield, and secondary modifications, which make tumor GS of FFPE material technically demanding [12]. Nevertheless, there is mounting evidence supporting the clinical usefulness and feasibility of FFPE tumor GS [13, 14, 15]. In these studies, FFPE tumor GS was either compared to panel analyses or to results from GS of FF samples; however, numbers of paired FFPE and FF samples were limited. Data on the diagnostic value of GS from FFPE tumors in the context of molecular tumor boards is scarce.

Here, we aimed to further investigate the technical and clinical applicability of FFPE tumor GS by a direct head‐to‐head comparison of sequencing results from FFPE tumor GS and ES. Thus, we generated matched ES and GS data of 100 ng of tumor DNA from FFPE tissue and corresponding normal DNA from peripheral blood for 15 cancer patients. We highlight potential pitfalls and provide recommendations for minimal requirements for successful tumor FFPE GS. In this real‐world scenario involving the type and quantity of available tumor tissue, we demonstrate that GS maintains its superior diagnostic performance compared to ES with an added diagnostic value in 40% (6/15) of patients.

## 2 Materials and Methods

### 2.1 Study Cohort

Our cohort comprised 15 patients, of whom 14 cancer patients were in an advanced disease stage and part of the prospective observational study *Molecular Tumor Board at the Center for Personalized Medicine Tübingen* (MTB@ZPM; NCT03503149) within the German Network for Personalized Medicine [1]. One patient had schwannomatosis, a rare disease defined by the development of benign nerve sheath tumors (ORPHA:93921) and CNA on Chr22. For a successful NGS, tissue specimens were fixed 6–24 h in neutral‐buffered 10% formalin and subsequently paraffin‐embedded according to routine diagnostic protocols. To be included, the amount of tumor DNA extracted from FFPE material had to be > 200 ng in order to have 100 ng of DNA for ES and GS, respectively. For Patient 8, DNA from two different tumor samples was used. The publication of this study was approved by the independent research ethics committee of the university hospital Tübingen (IED‐Project Number: 554/2025BO2).

### 2.2 DNA Isolation

Genomic DNA was isolated from normal tissue (peripheral blood) using Qiagen FlexiGene (Qiagen, Hilden, Germany). Tumor DNA was extracted from macrodissected 5 μm paraffin sections using the Maxwell RSC DNA FFPE Kit and the Maxwell RSC Instrument (Promega, Madison, WI, USA) according to the manufacturer's instructions. DNA concentrations were determined using the Qubit DNA BR kit (Thermo Fisher Scientific, Waltham, MA, USA), and DNA integrity was analyzed using the Agilent Genomic DNA Screen Tape System (Agilent, Santa Clara, CA, USA).

### 2.3 NGS Library Preparation

GS libraries were generated from 100 ng of peripheral blood DNA and FFPE tumor DNA using NEBNext UltraShear FFPE DNA Library Prep Kit (New England Biolabs) with 3 and 5 cycles of PCR amplification, respectively. Tumor‐normal DNA sample pairs of 15 patients were sequenced by GS and by exome sequencing (ES). For Patient 8, two different tumor samples were used. For all other samples, input DNA for ES and GS was identical. ES libraries were processed using the Library Preparation EF Kit v2 with a custom exome enrichment probe kit (Twist Bioscience). Libraries were sequenced in paired‐end mode (159+8+8+159) on an Illumina platform (NovaSeq6000 or NovaSeqXPlus).

### 2.4 Bioinformatic Analysis

Data processing was performed with our data analysis pipeline megSAP (https://github.com/imgag/megSAP). In brief, raw data were demultiplexed and converted into FASTQ data with Illumina bcl2fastq. Adapter sequences were removed with SeqPurge [16]. Mapping against the human reference genome (GRCh38) and variant detection were performed with Illumina DRAGEN. Variant annotation was performed using the variant effect predictor (VEP) and various damage scores. Germline and somatic copy number variation (CNV) calling was performed with ClinCNV (https://doi.org/10.1101/2022.06.10.495642). Manta [17] was used for the calling of structural variations (SV). Our decision support application GSvar (https://github.com/imgag/ngs‐bits) was used for variant interpretation and generation of final reports. Somatic variants were classified according to the Variant Interpretation for Cancer Consortium (VICC) [18]. Tumor mutation burden (TMB) was calculated based on Johnson et al. [19]. Homologous recombination deficiency (HRD) was determined using an HRD‐Score [20], which is the sum of somatic LOH, TAI, and LST alterations. An HRD‐Score > 42 was defined as HRD positive. Microsatellite instability (MSI) was analyzed with MSIsensor‐pro [21] and a cut‐off of > 10% was defined as MSI positive. Mutational signatures were extracted using SigProfilerExtractor (https://github.com/AlexandrovLab/SigProfilerExtractor) applied to variants called from FFPE‐derived GS and ES data. To mitigate potential FFPE‐related sequencing artifacts, variants with a VAF below 10% were excluded for GS samples.

### 2.5 Bioinformatic Validation Experiments (SNV/CNV Validation)

The validation of our ES and GS pipelines was done separately for small variants and copy number variants. The SNVs were validated using the GIAB reference samples HG001 and HG002. The samples were sequenced and 4 in silico tumor samples were generated by mixing 5%, 10%, 20%, and 40% of HG001 into the HG002 sample. Each mixture was mapped and the simulated somatic variants called using Illumina Dragen (v.4.3) in tumor‐normal calling mode with the pure HG002 sample as matched normal sample. The somatic variants were filtered to the high confidence region provided by GIAB (size: 2464 MB) and a minimum depth of 60× in both the HG002 sample and the mixture leaving a region of 2450 MB for validation. Variants occurring in HG001 as well as HG002 were removed from the comparison. The somatic variant calls were then validated against the GIAB reference calls of HG001 according to their expected allele fraction in the sample mixture.

CNVs were validated in our somatic target region of 1160 cancer‐related genes. Oncogenic CNVs detected in ES were used as a reference and for each CNV the difference of CNV detected by GS and CNV detected by ES was calculated. Homozygous deletions were counted as 0, heterozygous deletions as 1, copy‐neutral loss‐of‐heterozygosity as 2, amplifications as 3 or higher. Thus, exact matches had a difference (diff) of 0. For CNV callings with a diff greater than or less than 0, absolute values were used.

Sequencing coverage and quality statistics are listed in the supplement.

### 2.6 Evaluation of Potential Treatment Options

Patient histories and sequencing results were discussed at the locally established MTB [1, 22, 23]. In a first round, the MTB used the ES results for its recommendations, which were classified as NCT/ZPM evidence levels [24] and ESCAT tiers as described before [23]. In a second round (0.5–5 years later), the MTB used the GS results to identify any clinically relevant difference that changed the recommendation.

## 3 Results

### 3.1 Quality of Sequencing Data

In this single‐center study, we sequenced 100 ng of FFPE tumor DNA from a cohort of 15 cancer patients by ES and GS. Our cohort consisted of different tumor types (melanoma *n* = 4, rectal cancer *n* = 3, breast cancer *n* = 2, pancreatic adenocarcinoma *n* = 1, intestinal ampullary carcinoma *n* = 1, papillary thyroid cancer *n* = 1, cervical adenocarcinoma *n* = 1, peritoneal mesothelioma *n* = 1, schwannoma *n* = 1) and FFPE tumor samples had a tumor content ranging from 10% to 90% (Figure S1).

First, we evaluated the technical/sequencing performance of GS compared to ES (Figure 1). Mapping quality was similar between ES and GS (Figure 1a). GS data was of slightly poorer quality with a reduction in insert size and Q30 base percentage (Figure 1b,c) as intronic sequences contain more homopolymeric tracts, which are prone to sequencing errors. Median coverage was 110 and 36 in GS and 353 and 195 in ES for tumor and normal samples, respectively (Figure 1d). Coverage heterogeneity was altered in GS compared to ES, showing a reduced AT dropout with an increase in GC dropout (Figure 1e,f). This underrepresentation of genomic regions with a high GC content was most prominent in tumor GS samples.

**FIGURE 1:**
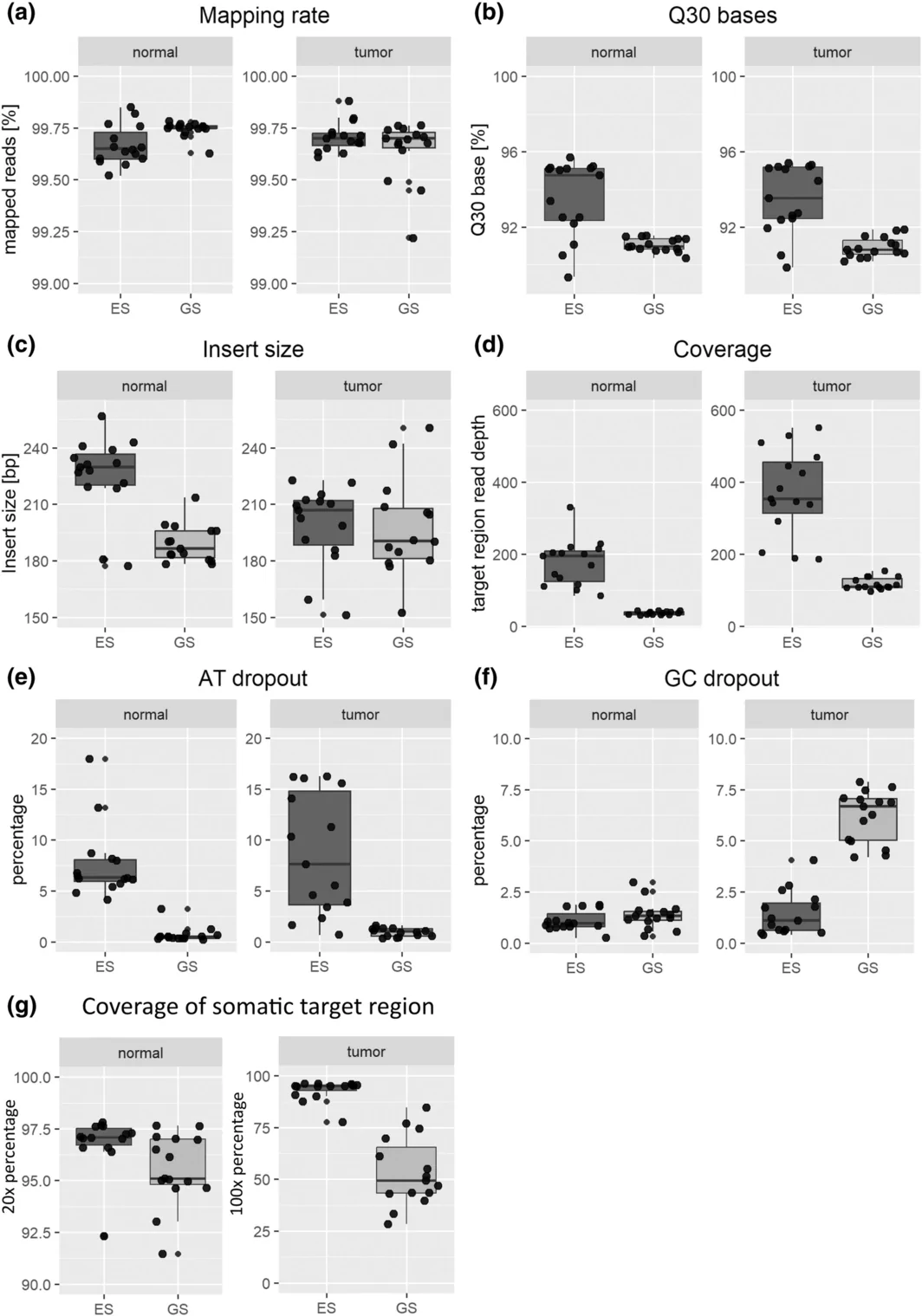
Comparison of sequencing quality metrics. (a) Mapping rate was similar in ES and GS protocols. (b) Percentage of bases with high quality (quality score ≥ 30) was slightly reduced in GS. (c) Insert size was lower in GS protocol. (d) Coverage was reduced in GS compared to ES since a mean coverage of 100× and 30× was aimed for in tumor and in normal samples, respectively. (e) AT dropout was elevated in ES compared to GS. (f) GC dropout was strongly increased in tumor GS samples compared to ES samples. (g) Minimal coverage of 20× was achieved in normal GS samples for an average of 95% of the somatic target region. Minimal coverage of 100× was achieved in tumor GS samples for an average of 49% of the somatic target region.

From a diagnostic point of view, a uniform coverage of cancer‐related genes is important for reliable detection of somatic small nucleotide variants (SNV). A mean coverage of 100× was reached in an average of 49% of our target region of 1160 cancer‐related genes (4.6 Mb) in all tumor GS samples (Figure 1g). In normal samples, 95% of our somatic target region had a sequencing depth of at least 20 in GS sufficient for reliable detection of germline variants (Figure 1g). We further evaluated regions with extremely low coverage of < 20 reads in tumor samples as these are prone to introduce gaps in SNV detection. Consistent with the high GC‐dropout rate, GC‐rich regions were most commonly affected, potentially due to PCR amplification of the input material. Low‐coverage regions that were present in more than 50% of samples constituted 1.5% and 1.6% of our somatic target region in tumor and normal samples, respectively (data not shown). For example, the *TERT* promotor region at position c.‐124 and c.‐146 was strongly affected with a coverage of < 20 reads in 80% (12/15) and 67% (10/15) of tumor samples, respectively (data not shown). Bioinformatic validation of SNV detection showed a sensitivity of > 95% and precision of > 99% at an allele fraction of 10% for both GS and ES (Table S1). Sensitivity of INDEL detection was equally reduced (Table S1).

### 3.2 Diagnostic Utility of GS Analysis

Due to the differences in sequencing quality, target region and coverage between GS and ES, we thoroughly compared whether GS can be used instead of ES in a diagnostic setting within an established MTB structure. First, we examined the variant allele frequencies (VAF) of all small variants detected by GS and/or ES for each patient (Figure S2). There was a good VAF correlation of concordant small variants for the two system types. However, a substantial number of small variants were only detected by either of the two sequencing systems. Therefore, we further examined these unique variants by variant types (Figure S3). The majority of small variants detected only by one assay occurred at low VAF (< 10%) and did not represent diagnostically relevant variant classes (i.e., exonic variant types: missense, insertion‐deletions (indel), stop, frameshift, or canonical splice site) or genes (Figure S3a,b). This may reflect the fact that GS captures additional intronic small variants, but misses small variants with a low allele fraction. Looking only at cancer‐related genes, we observed a high rate of missense and indels that only occurred in ES (Figure S3c). These were subclonal variants with a VAF < 10% since setting a VAF threshold required for diagnostic reporting (i.e., VAF > 10%) removed the majority of these variants (Figure S3d). Yet, GS was not able to fully capture all missense and indel small variants from ES even with a high VAF. This was due to a reduced coverage of < 20 reads in GS at the respective genomic positions.

To further investigate whether GS is able to capture diagnostically relevant SNVs, we evaluated SNV recall in GS by using SNVs detected in ES as a reference (Figure 2a, Table S2). Taking into account all SNVs in cancer‐related genes, a 100% SNV recall in GS was only achieved in 3 out of 15 patients. From a diagnostic point of view, likely oncogenic/oncogenic SNVs are most relevant as they are likely the main tumor drivers and potential therapeutic targets. When evaluating only likely oncogenic and oncogenic SNVs, both analyses showed a better concordance in SNV detection. Nevertheless, in 6 out of 13 patients with oncogenic SNVs some variants were not detected in the initial GS analysis due to low coverage at the respective genomic locus. Therefore, we refined our filtering criteria to include variants with a coverage of < 20 in tumor or normal samples. With this GS specific filter, we were able to correctly identify all oncogenic SNVs in 11 out of 13 patients. The two oncogenic variants that were still missing were located in the *TERT* promotor and could not be identified due to extremely low coverage of 3 and 6 reads at these positions.

**FIGURE 2:**
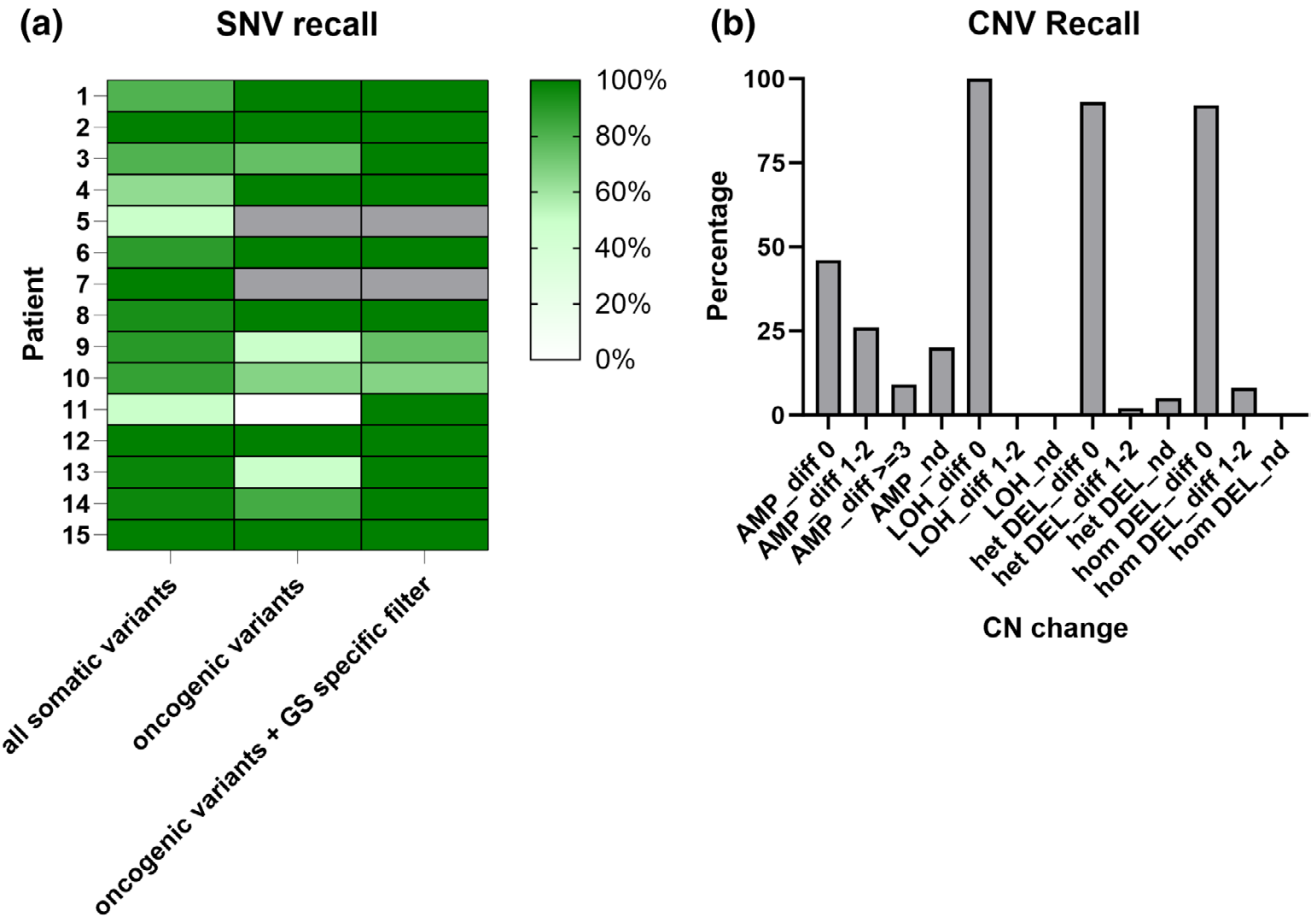
Assessment of diagnostic detection of single nucleotide variants (SNVs) and copy number variations (CNVs). In both analyses, diagnostically relevant alterations from ES data were used as reference. (a) SNV detection showed a 100% concordance in 4 out of 15 patients for all SNVs in our somatic target region. Reducing the reference data set to likely oncogenic or oncogenic SNVs resulted in a complete recall in 7 out of 13 patients. We further adapted our filtering settings and introduced a genome sequencing (GS) specific filter that includes variants with a coverage below 20 reads at the respective genomic position. With this GS specific filter a 100% diagnostic SNV recall of oncogenic SNVs was achieved in 11 out of 13 patients. Gray fields, no variants detected in ES. (b) CNV recall was analyzed as numeric difference (diff) of the detected copy number alteration in GS versus ES. Exact matches (i.e., diff 0) of CNVs were achieved in the majority of detected CNVs. Amplifications (AMP) showed the highest variation and 16% of amplifications were not detected in GS. Copy number calling of copy‐neutral loss‐of‐heterozygosity (LOH), heterozygous deletions (het DEL) and homozygous deletions (hom DEL) showed a concordance of > 90%. One homozygous deletion in ES was determined as heterozygous by GS. Nd, not detected.

In a similar approach, we evaluated whether copy number variations (CNVs) of diagnostically relevant genes were correctly detected in GS (Figure 2b). GS was able to identify 44% (67/154) of amplifications (AMP), 98% (98/108) of heterozygous deletions (het DEL), 94% (14/15) of homozygous deletions (hom DEL), and 100% (3/3) of copy‐neutral loss‐of‐heterozygosity (LOH). In case of amplifications, a minor deviation of 1 or 2 from the original copy number is also acceptable, which added another 29% (45/154). GS did not detect 16% of amplifications; however, these were mainly subclonal with a clonality of < 20% (data not shown). High differences (diff ≥ 3) in amplifications were mostly due to assignment of different clonalities in ES and GS data (data not shown). Further, one homozygous deletion was determined heterozygous by the CNV analysis pipeline in GS. Yet, it was correctly identified as a homozygous deletion via the SV calling.

Complex biomarkers were also compared between GS and ES analysis (Figure 3). Overall, values for TMB, HRD, and MSI showed a good positive correlation (Spearman correlation coefficient of 0.98, 0.85, and 0.89, respectively). Yet, for TMB and HRD detection, some general bias was observed. TMB was slightly, but significantly, increased in GS analysis, possibly due to the expanded target region (Figure 3a). HRD Score was significantly reduced in GS (Figure 3b). This discrepancy might be explained by the high number of subclonal CNV calls in ES analysis, which was greatly reduced in GS analysis. Patient 8 in our cohort with triple‐negative breast cancer served as a positive control for HRD detection as she had benefitted from PARP inhibitor treatment and was determined HRD positive (HRD Score 51) in ES. GS also detected a positive HRD score of 55 for this patient, suggesting sustained sensitivity of GS in HRD detection. Values for MSI detection were not significantly different; however, there was no MSI‐positive patient in our cohort (Figure 3c). Mutation signatures showed cancer‐type specific signatures in 2 samples in ES and in 4 samples in GS (Figure 3d,e). Signatures associated with UV‐exposure (SBS7a,b) were detected in all three patients with skin melanoma in GS and only in two of these patients in ES. An HRD‐related signature (SBS3) was detected in the HRD‐positive TNBC patient by GS only. This suggests an enhanced detection of mutation signatures in GS compared to ES; however, our cohort size was too small for correlation analysis of detected signatures and associated tumor properties.

**FIGURE 3:**
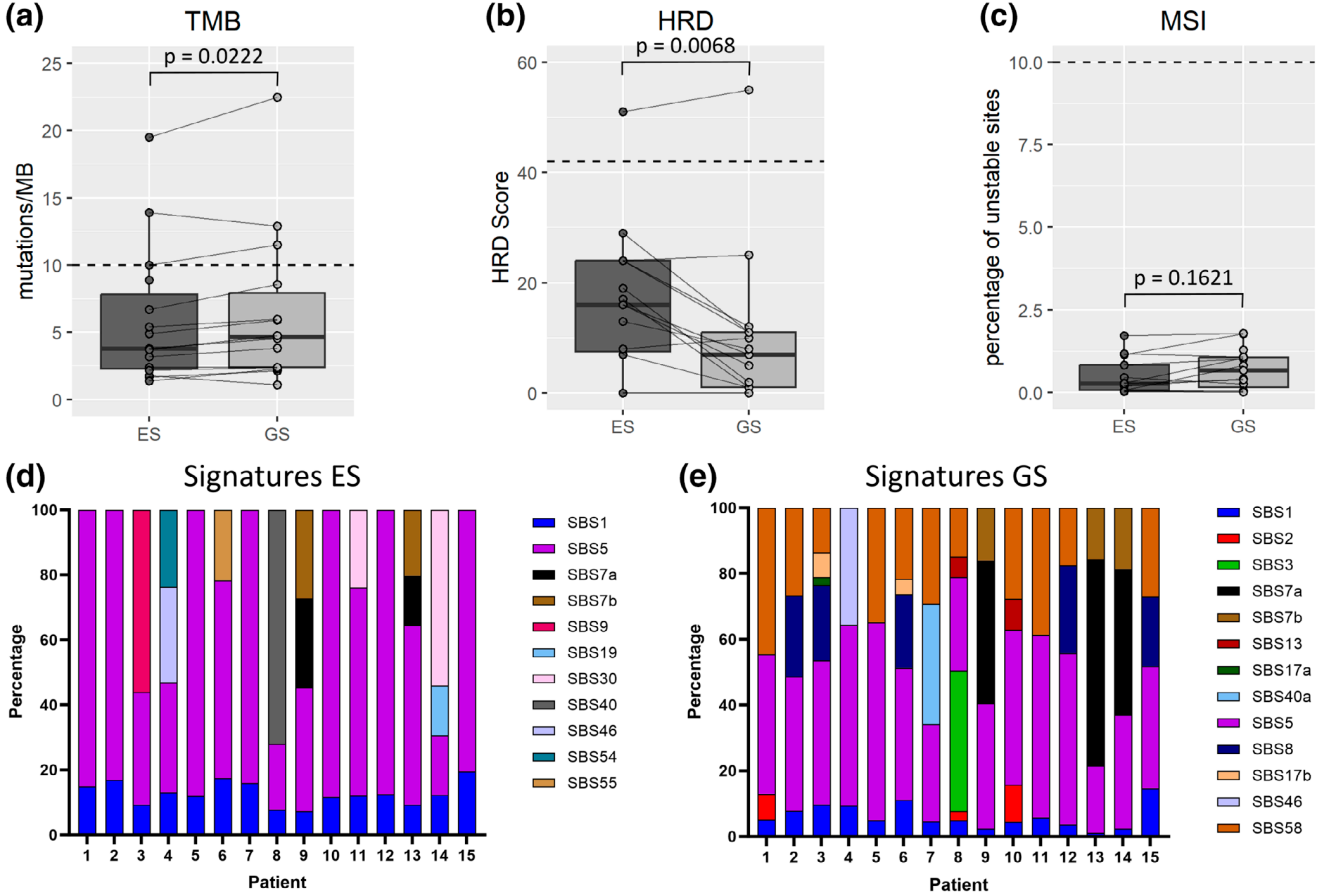
Detection of complex biomarkers. (a) Tumor mutation burden (TMB) was slightly, but significantly enhanced in GS. GS and ES consistently detected high TMB (> 10 mutations/Mb) in three patients. (b) Homologous recombination deficiency (HRD) was assessed by calculation of an HRD Score. It was significantly reduced in GS compared to ES potentially due to differences in CNV detection. One patient was determined HRD‐positive (HRD Score > 42) in both analyses. (c) Microsatellite instability (MSI) status was not significantly altered. (d) COSMIC mutation signature composition for each sample derived from exome sequencing data. Sample 9 and 13 showed specific UV‐related signatures SBS7a and SBS7b. (e) COSMIC mutation signature composition for each sample derived from genome sequencing data. Sample 9,13, and 14 showed specific UV‐related signatures SBS7a and SBS7b. Sample 3 compromised defective homologous recombination signature SBS3. Dashed lines in a–c: diagnostic cut‐off for each biomarker; significance test in a–c: Wilcoxon matched‐pairs signed rank test.

### 3.3 Added Diagnostic Value of GS Analysis

Diagnostic evaluation of both analyses showed an additional oncogenic alteration in 6 out of 15 patients in GS (Table 1, Table S3). This was due to enhanced detection of SVs and CNVs of focal deletions in GS. Newly identified alterations comprised focal homozygous deletions in *PTEN* (*n* = 2), *CDKN2A* (*n* = 1), *MAP2K4* (*n* = 1), *BRAF* (*n* = 1), and *ARID1B* (*n* = 1). The detected alterations affected various signaling pathways, which translated into new treatment options for these patients upon evaluation of the local molecular tumor board.

**TABLE 1:** Additional oncogenic alterations and clinical recommendations. In 6 out of 15 samples an additional oncogenic alteration was identified by enhanced detection of copy‐number variations (CNVs) or structural variations (SVs).

Patient	Tumor type	Alteration type	Additional alterations	Biological implication	Treatment recommendation	Impact on treatment priorities
2	PAAD	SV/CNV	*MAP2K4* (hom DEL/translocation), *ARID1B* (hom DEL)	MEK‐ERK signaling pathway activation	*MAP2K4*: MEK inhibition, e.g., trametinib, selumetinib, binimetinib (NCT/ZPM evidence m3; ESCAT Tier V) *ARID1B*: no recommendation	Recommendation of first priority further supported
7	MUP	SV	*MAP3K8‐PARD3* (inversion), *BRCA1*(2 inversions)	MEK–ERK signaling pathway activation, potential HRD	*MAP3K8‐PARD3*: MEK inhibition, e.g., trametinib, selumetinib, binimetinib (NCT/ZPM evidence m3; ESCAT Tier V) *BRCA1*: Olaparib + Pembrolizumab (NCT/ZPM Evidence m2C)	New third priority
8	TNBC	SV	*JAK1‐SETD2* (translocation), *SETD2* (translocation), NBN (translocation)	HRD	*NBN*: Olaparib + Ceralasertib (NCT/ZPM evidence m1C) *SETD2*: Camonsertib (clinical trial NCT 04589845)	Prior recommendation supported by identification of somatic driver of HRD
9	SKCM	SV/CNV	*PTEN* (hom DEL), *BRAF* (hom DEL exon 3–10), *ATR* (5 inversions)	MTOR signaling pathway activation, alternative BRAF product, potential HRD	*BRAF*: Resistance to BRAF inhibition *PTEN*: Capivasertib + Paclitaxel (NCT/ZPM evidence m2C), resistance to T cell‐mediated immunotherapy [25] *ATR*: no recommendation	Loss of priority 1 (resistance detected), new first priority
12	PEMESO	SV	*CDKN2A* (hom DEL)	Cell cycle activation	*CDKN2A*: Abemaciclib (NCT/ZPM evidence m1C)	Additional support for recommended third priority
13	SKCM	SV	*PTEN* (hom DEL)	MTOR signaling pathway activation	*PTEN*: Capivasertib + Paclitaxel (NCT/ZPM evidence m2C), resistance to T cell‐mediated immunotherapy [25]	New first priority

*Note:* Additional alterations compromised homozygous deletions in *PTEN* (*n* = 2), *CDKN2A* (*n* = 1), *MAP2K4* (*n* = 1), *BRAF* (*n* = 1), and *ARID1B* (*n* = 1). One inversion was predicted to generate an oncogenic fusion in MAP3K8. HRD‐related genes, that is, *BRCA1*, *SETD2*, *NBN*, and *ATR* were affected by several SVs. These alterations led to activation of different signaling pathways or were associated with a potential HRD in these tumors. These alterations were translated into treatment recommendations by the local molecular tumor board. Existing treatment recommendations were supported in three patients, an additional treatment option was suggested in three patients and in one patient a resistance to a recommended treatment was detected.

Abbreviations: hom DEL, homozygous deletion; MUP, melanoma of unknown primary; PAAD, pancreatic adenocarcinoma; PEMESO, peritoneal mesothelioma; SKCM, cutaneous melanoma; TNBC, triple‐negative breast cancer.

HRD was suggested in three patients due to additional detection of translocations or multiple inversions in HRD‐related genes, namely *BRCA1*, *SETD2*, *NBN*, and *ATR*. In one of these patients (Patient 8), a positive HRD‐Score was detected as well. Taken together, these alterations supported the prior recommendation of PARP inhibitor treatment in this patient.

Furthermore, in a BRAF‐negative melanoma of unknown primary (Patient 7) GS was able to identify the missing oncogenic driver of this tumor, an activating *MAP3K8* fusion. This translated into a new treatment option with a MEK‐inhibitor (e.g., Trametinib, Binimetinib, Selumetinib) as third priority with an evidence level of m3 (ESCAT Tier V) (Table 1).

In a *BRAF* V600E driven melanoma (Patient 9) a focal deletion of *BRAF* exons 3–10 was detected. Internal *BRAF* deletions have been described as a resistance mechanism to BRAF inhibitor treatment [26]. Consequently, in our case, the former first treatment priority of BRAF/MEK inhibition was dropped.

Taken together, additional alterations detected by GS lead to new recommendations with high priority [1, 2, 3] in three patients. In three patients, the existing recommendations were further supported by the detected molecular alterations.

## 4 Discussion

FFPE material has long been considered not suitable for GS, limiting its widespread application in tumor diagnostics. In this study, we present data from FFPE tumor GS with an input of 100 ng of DNA. Direct comparison of obtained GS data to matched ES data allowed a close technical and clinical evaluation. Therefore, we could identify potential pitfalls in GS from FFPE material and suggest some minimum requirements for tumor GS application and data interpretation. Importantly, due to the superiority of GS in SV detection, additional alterations were identified in 40% (6/15) of cancer patients. MTB evaluation of these alterations highlighted their clinical importance and impact on patient management.

In general, the quality of the obtained sequencing data was sufficient for reliable SNV detection comparable to ES analysis. As both tumor analyses were performed with the same DNA from FFPE material, we did not evaluate the content of FFPE artifacts in our samples. QC parameters indicated a nonuniform coverage as reported by others for FFPE material [27, 28, 29], which was enhanced in our GS cohort compared to ES. This may be attributed to the unspecific amplification of input DNA by polymerase chain reaction in the GS protocol in contrast to enrichment of underrepresented regions in the ES protocol.

This loss in coverage uniformity mostly affected GC‐rich regions and led to coverage gaps, including the diagnostically relevant *TERT* promotor as described before for FFPE tumor GS [14]. This issue can be indirectly resolved by adapting variant filtering criteria to include low coverage regions. However, this strategy was not able to rescue variants in regions with an extremely low coverage. Thus, negative sequencing results for oncogenic variants in the *TERT* promotor should be interpreted/reported with caution taking into account the sequencing depth at the respective genomic positions. In general, this example highlights the necessity of technical improvements in these hard‐to‐sequence regions.

We also observed general discrepancies in SNV detection between the two sequencing systems. The majority of SNVs unique to one analysis had a VAF below 10%, consistent with a drop in sensitivity in SNV validation with a VAF cut‐off of lower than 10%, especially for GS analysis. Therefore, we recommend setting a cutoff of 10% VAF for reporting diagnostic variants. Together with the reduced average sequencing depth in GS, this highlights the need for sufficient tumor content for reliable detection of somatic SNVs. This is illustrated by a loss of 50% (4 out of 8 SNVs detected) of somatic SNVs in patient 11 with a tumor content of 10%. All other samples showed sufficient SNV recall in GS, suggesting that a tumor content of at least 30% is required for tumor GS. This is in line with recommendations from larger studies, which advise using a minimal amount of 20%–30% TC in combination with 100× read coverage [30, 31].

When evaluating HRD detection in GS and ES, we observed a significant reduction of the HRD Score in GS. The HRD Score is based on CNV calling in those datasets. Bioinformatic analysis of CNVs is technically different in ES and GS due to differences in the target regions. This leads to a higher fragmentation of CNV calls in ES, which cannot easily be resolved as the majority of CNV breakpoints are not covered in ES analysis. In general, the applicability of the HRD Score, which was originally developed on SNP array data, in ES and GS has been shown previously [32, 33]. In line with these studies, the HRD positive patient in our cohort had a high HRD Score (> 42) in both ES and GS. However, de Luca et al. also show some patients may be differentially classified by different assays and suggest including further evidence, that is, mutations signatures or HR gene mutation status to determine HRD status. In this regard, we observed in our study that GS is able to detect more specific mutation signatures than ES. Nonetheless, further evaluation in a larger tumor cohort is required to define definitive HRD score and TMB cut‐offs using genomic sequencing.

From our experience of tumor FFPE GS in this small cohort, several technical requirements can be derived. First, DNA fragmentation should be optimized in order to yield insert sizes of 250 bp length as recommended by Xiao et al. [30]. In this study, 200 ng of FFPE DNA had to be available to perform matched ES and GS analysis of the same specimen. This may introduce a bias towards specimens with better DNA quality. However, application and validation of our GS protocol for accreditation in a diagnostic setting yielded similar results for 50 ng of input DNA (data not shown). The amount of 100 ng of DNA as starting material requires PCR amplification before library preparation. Yet, PCR amplification seemed to enhance AT/GC bias and should therefore be kept at a minimum. Here, we used 5 and 3 cycles for tumor and normal DNA respectively. Furthermore, the observed high amount of GC dropout suggests optimizing the annealing temperature during PCR to improve coverage uniformity. To detect variants with VAF as low as 10%, a minimal sequencing depth of 100× for tumor DNA and 30× for normal DNA should be achieved. Here, 2500 × 10^6^ sequencing clusters were used per tumor‐normal pair. For diagnostic purposes we increased the cluster numbers and achieved 100× coverage in 85% of our target region in tumor samples. As no paired analysis was done, we do not include these GS in this report.

As GS covers all intronic regions, breakpoint detection of SVs and CNVs is greatly enhanced compared to ES [3, 5]. This is reflected in our study and paves the ground for GS outperforming ES in detecting therapeutically relevant alterations. In this small cohort, we detected additional oncogenic alterations in 40% (6/15) of patients due to an enhanced detection of focal homozygous deletions and SVs. Clinical assessment of these alterations by the locally established MTB showed their high actionability. Hence, existing treatment options were supported in 20% (3/15) of patients, resistance to targeted treatment was detected in 7% (1/15) of patients, and new treatment options were available for 20% (3/15) of patients. This is comparable to results from a study conducted in 78 melanoma patients, in which comparison of GS to panel analysis yielded 38% additional actionable markers and 18% of new treatment options [14]. Pre‐selection of patients lacking an oncogenic driver identified by ES substantially increased these rates to 68.4% and 22.6% for the identification of driver or likely driver alterations by GS, respectively [31]. This is reflected in our Patient 7, in whom we were able to identify the missing oncogenic driver and underscores a strong superiority of GS compared to ES in such cases. In a larger study of GS analysis of > 700 tumors from FF material, comparison to in silico ES or panel analysis showed an increase in detection of biomarkers for clinical trials [7]. Detection rates of biomarkers for approved drugs were only marginally increased in GS compared to ES. However, the authors acknowledge that in silico down‐sampling of GS data may not fully reflect potential biases/technical shortcomings of real‐world ES.

In summary, our head‐to‐head comparison of GS and ES in a cohort of patients with different cancer types indicates that tumor GS from FFPE material is a reliable tool to detect tumor variants. GS identified additional clinically actionable alterations compared to ES. This argues for its routine implementation in clinical diagnostics for molecular tumor boards as it has the potential to substantially enhance personalized treatment decisions in cancer patients. The usability of GS in personalized medicine is currently evaluated in large national initiatives like the German “Model Project Genome Sequencing.” The current study provides guidance for the implementation of tumor GS and its implementation in personalized cancer care.

## Author Contributions

**Nadja Ballin:** visualization, formal analysis, data curation, writing – original draft. **Alexander Ott:** formal analysis, methodology. **Michaela Pogoda:** methodology. **Florian Harmuth:** methodology. **Vijay Kumar Ulaganathan:** formal analysis. **Anton Karelin:** formal analysis. **Cristiana Roggia:** writing – review and editing. **Irina Bonzheim:** methodology. **Stephan Singer:** methodology. **Holly Sundberg‐Malek:** formal analysis. **Pavlos Missios:** writing – review and editing. **Stephan Ossowski:** formal analysis, methodology. **Tobias B. Haack:** writing – review and editing, resources. **Olaf Riess:** writing – review and editing, resources. **Michael Bitzer:** writing – review and editing, investigation. **Nisar Malek:** writing – review and editing. **Christopher Schroeder:** conceptualization, methodology, supervision, writing – review and editing. **Sorin Armeanu‐Ebinger:** conceptualization, methodology, writing – review and editing, supervision.

## Funding

This work was supported by Deutsche Forschungsgemeinschaft, 286/2020B01—428994620.

## Ethics Statement

The publication of this study was approved by the independent research ethics committee of the university hospital Tübingen (IED‐Project Number: 554/2025BO2).

## Consent

Adult patients were evaluated for this pilot study based on the indication for comprehensive molecular profiling by the oncology tumor board and after the informed consent process. The publication of this study was approved by the independent research ethics committee of the university hospital Tübingen (IED‐Project Number: 554/2025BO2).

## Conflicts of Interest

The authors declare no conflicts of interest.

## Supporting information


**Figure S1:** Study design and study cohort of 15 patients. The cohort compromised different tumor types with a tumor content (TC) in the analyzed tumor material from 10% to 90%. 100 ng of tumor and normal DNA, respectively, were sequenced by ES and GS. After sequencing, bioinformatic analysis, analyst curation, and evaluation of the clinical report in a molecular tumor board was performed. This allowed a head‐to‐head comparison of technical QC parameter, diagnostic parameters like SNV and CNV recall, SV and biomarkers as well as an assessment of the diagnostic value of GS compared to ES. Tumor entities are indicated as Oncotree Codes. *TNBC, triple‐negative breast cancer.
**Figure S2:** Variation of variant allele frequencies (VAF) between ES and GS. Pearson correlation was plotted for variants detected by both sequencing systems. All samples contained variants detected either by ES or GS‐only. These were more abundant in GS samples.
**Figure S3:** Detection of different types of SNVs via ES and GS. ES‐unique and GS‐unique SNV types were divided into diagnostically relevant categories of exonic missense and insertion deletion (indel), stop and frameshift, canonical splice site variants. The remaining variants were summarized as “other” and included mostly intronic variants. (a) An unfiltered overview on all SNVs detected only by one sequencing system. (b) SNV types after filtering for an AF > 10% show same distribution. (c) SNV types in target region (i.e., cancer‐related genes) contain a high amount of ES‐unique missense & indels. (d) additional filtering of SNVs in the target region with an AF > 10% strongly reduces ES‐unique SNVs. The majority of missed SNVs are GS‐unique in the diagnostically less relevant category “other.” A discrepancy of relevant SNV detection remains in missense and indels. Absolute numbers of SNVS are indicated on top of each bar.

**Table S1:** Bioinformatic validation of SNV detection.
**Table S2:** Detection of diagnostically relevant small variants. In each patient, small variants in cancer‐related genes identified by ES were verified in GS. Absolute numbers are indicated for GS and for ES in brackets. The same analysis was performed for likely onocogenic and onocgenic small variants. This was the basis for definition of successfull oncogenic recall. Here, in 7 out of 13 patients with oncogenic small variants a complete detection of these small variants by GS was achieved. Upon adatped filtering settings to include also genomic loci with low sequencing depth (< 20) oncogenic recall was assessed again. This lead to an increase in successfull recall of oncogenic small variants by GS to 11 out 13 patients. n/a, no oncogenic small variants detected by ES; # sequencing depth < 6 at small variant position with undetected small variant in GS; TC, tumor content.
**Table S3:** Detailed description of additional alterations and evidence for clinical recommendations.

**Table S4:** Sequencing covarage and quality statitics.

## Data Availability

The datasets generated and analyzed during the current study are not publicly available due to the German Genetic Diagnostics Act. Parts of it can be shared upon reasonable request to the corresponding author.
