# Supplementary material for: Genome Sequencing Outperforms Exome Sequencing in Formalin‐Fixed Paraffin Embedded Tumors Leading to Improved Treatment Options
Source: Cancer Med. 2026 Oct 27;15(11):e72448. doi: 10.1002/cam4.72448 (PMC13714339; doi:10.1002/cam4.72448)
Supplement: Supplementary file 1 [file CAM4-15-e72448-s001.docx]

| **Sample ID** | **Total number of sequencing reads** | **Total number of uniquely mapped non-duplicate reads (GRCh38)** | **Total number of covered bases** | **Median coverage (and range) per base** | **Percentage of targeted bases with coverage >= 10** |
| --- | --- | --- | --- | --- | --- |
| T1 | 365143727 | 276232994 | 49370739 | 463 (0-18390) | 97.94 |
| T2 | 397220033 | 300962945 | 49331317 | 492 (0-22013) | 97.83 |
| T3 | 254108310 | 211524621 | 49348305 | 330 (0-10466) | 97.68 |
| T4 | 300551462 | 230354894 | 49381710 | 378 (0-16620) | 97.84 |
| T5 | 223191415 | 173038528 | 49253851 | 169 (0-5272) | 97.47 |
| T6 | 244541507 | 192531088 | 49351107 | 177 (0-6796) | 97.66 |
| T7 | 314820737 | 250020930 | 49318815 | 418 (0-15024) | 97.77 |
| T8 | 336191270 | 230069533 | 49231926 | 196 (0-4341) | 97.22 |
| T9 | 383516516 | 280316806 | 49346112 | 251 (0-9719) | 97.86 |
| T10 | 327012143 | 269894473 | 49367432 | 420 (0-13368) | 97.80 |
| T11 | 271992385 | 225378638 | 49279296 | 345 (0-9510) | 97.57 |
| T12 | 260140081 | 214721344 | 49296589 | 329 (0-10698) | 97.63 |
| T13 | 383907434 | 292424253 | 49359548 | 486 (0-21061) | 97.83 |
| T14 | 279258181 | 205590715 | 49177030 | 310 (0-14024) | 97.21 |
| T15 | 324144814 | 251793539 | 49285989 | 431 (0-14356) | 97.70 |
| N1 | 147675251 | 114404523 | 49343157 | 187 (0-8255) | 97.70 |
| N2 | 162779955 | 130208693 | 49292039 | 220 (0-11264) | 97.64 |
| N3 | 151228778 | 122299423 | 49316408 | 134 (0-3877) | 97.64 |
| N4 | 138969336 | 115367638 | 49326089 | 199 (0-8961) | 97.68 |
| N5 | 110185150 | 89398696 | 49260723 | 92 (0-2600) | 95.32 |
| N6 | 96955531 | 82191052 | 49293209 | 80 (0-3895) | 97.24 |
| N7 | 246596309 | 200336379 | 49399913 | 325 (0-10313) | 97.88 |
| N8 | 146259214 | 115677771 | 49094936 | 108 (0-2835) | 96.90 |
| N9 | 176748915 | 137385703 | 49145815 | 130 (0-4448) | 97.18 |
| N10 | 147484866 | 127320785 | 49333083 | 197 (0-7442) | 97.54 |
| N11 | 125663471 | 108119489 | 49300157 | 114 (0-3925) | 97.14 |
| N12 | 125327299 | 109503965 | 49280832 | 165 (0-6099) | 97.36 |
| N13 | 158937379 | 126189416 | 49375639 | 211 (0-11739) | 97.82 |
| N14 | 150338868 | 118909285 | 49355782 | 197 (0-9681) | 97.74 |
| N15 | 158651786 | 124467662 | 49322914 | 207 (0-8397) | 97.70 |
| T1 | 4557378336 | 3777756638 | 2861150267 | 194 (0-539727) | 96.99 |
| T2 | 4477270594 | 3695088440 | 2861117021 | 190 (0-683479) | 96.94 |
| T3 | 4055355936 | 3415309086 | 2852556036 | 161 (0-289891) | 96.65 |
| T4 | 4227496600 | 3412639156 | 2854943281 | 156 (0-208123) | 96.77 |
| T5 | 3894567254 | 3219700584 | 2869258092 | 161 (0-274616) | 97.26 |
| T6 | 4392425372 | 3712392605 | 2866687668 | 176 (0-184449) | 97.22 |
| T7 | 4213394650 | 3506626812 | 2849307264 | 169 (0-181104) | 96.62 |
| T8 | 4836549968 | 4039639234 | 2843286168 | 182 (0-146058) | 96.47 |
| T9 | 3804904782 | 3211690518 | 2869806069 | 152 (0-298361) | 97.26 |
| T10 | 3552890330 | 3020122777 | 2866885876 | 153 (0-250919) | 97.18 |
| T11 | 3570213068 | 3068319690 | 2848122864 | 151 (0-206759) | 96.53 |
| T12 | 4772174968 | 4018637562 | 2852581590 | 199 (0-211804) | 96.70 |
| T13 | 5852225966 | 4832155761 | 2869990659 | 224 (0-206661) | 97.36 |
| T14 | 5516026344 | 4550868323 | 2855184884 | 185 (0-153073) | 96.93 |
| T15 | 4787977832 | 4007600610 | 2856847637 | 199 (0-381090) | 96.87 |
| N1 | 1372223729 | 1191501324 | 2842293164 | 59 (0-68001) | 96.25 |
| N2 | 1301487003 | 1133407322 | 2840527584 | 56 (0-62535) | 96.17 |
| N3 | 1019778268 | 899150235 | 2840977393 | 45 (0-85103) | 95.97 |
| N4 | 1107898159 | 982897903 | 2837343902 | 48 (0-67516) | 96.03 |
| N5 | 1220396308 | 1085478859 | 2849738526 | 54 (0-64986) | 96.20 |
| N6 | 1341061429 | 1181669467 | 2854006892 | 58 (0-65779) | 96.62 |
| N7 | 1208022241 | 1061019096 | 2837818883 | 52 (0-56361) | 96.05 |
| N8 | 1100620332 | 950650206 | 2837782825 | 46 (0-60563) | 96.01 |
| N9 | 1477554044 | 1285698610 | 2855217042 | 63 (0-71350) | 96.67 |
| N10 | 1367872111 | 1198019847 | 2853495523 | 58 (0-60291) | 96.60 |
| N11 | 1178094395 | 1044588006 | 2838114930 | 51 (0-64537) | 96.03 |
| N12 | 1489615558 | 1301815596 | 2841299274 | 65 (0-66857) | 96.27 |
| N13 | 1464213843 | 1273988241 | 2856558645 | 63 (0-66650) | 96.75 |
| N14 | 1491436070 | 1302231575 | 2857730418 | 66 (0-69151) | 96.81 |
| N15 | 1581671575 | 1377687642 | 2842812106 | 67 (0-75276) | 96.29 |

Table S4: Sequencing Covarage and Quality Statitics
